# T-cell recognition of SARS-CoV-2 antigen peptides following booster vaccination with mRNA and viral-vectored COVID-19 vaccines

**DOI:** 10.3389/fcimb.2026.1947651

**Published:** 2026-10-19

**Authors:** Adams Kiplimo Maru, Precious Kwesi Kumi, Abigail Naa Adjorkor Pobee, Nana Yaa Asiedua Appiah, Nana Akua Obiribea Koranteng, Theophilus Brenko, Frank Osei, Doreen Teye-Adjei, Georgina Agyekum, Linda Akuffo, Francis Dzabeng, Eric Kyei-Baafour, Dorcas Obiri-Yeboah, Yaw Aniweh, Michael Fokuo Ofori, Kwadwo Asamoah Kusi, Frederica Dedo Partey

**Affiliations:** 1 Department of Immunology, Noguchi Memorial Institute for Medical Research, College of Health Sciences, University of Ghana, Accra, Ghana; 2 Department of Microbiology and Immunology, School of Medical Sciences, University of Cape Coast, Cape Coast, Ghana; 3 West African Centre for Cell Biology of Infectious Pathogens, College of Basic and Applied Sciences, University of Ghana, Accra, Ghana

**Keywords:** COVID-19, SARS-CoV-2 epitopes, vaccines, booster shot, immune response, CD8+ T cell epitopes, cellular vaccine immune responses

## Abstract

**Background:**

Durability and breadth of SARS-CoV-2-specific T-cell responses following booster vaccination remain key to guiding next-generation vaccine design, especially in the context of waning antibodies, emerging variants, and diverse immunological backgrounds. This longitudinal study assessed IFN-γ-producing T-cell responses in 46 fully vaccinated adults who received a booster dose of either the BNT162b2 (mRNA) or Ad26.COV2.S (adenoviral vector) vaccine. All participants were nucleocapsid-seropositive at baseline, with antibody levels stable throughout follow-up, indicating prior natural infection and no incident infection during the study period.

**Methods:**

Using an ex vivo IFN-γ ELISpot assay, responses were measured at four timepoints (baseline, Months 3, 6 and 9) against spike (S; n = 46) and nucleocapsid (N; n = 16) peptides selected by NetMHCpan-4.1 prediction from overlapping 15-mer libraries spanning both proteins. Each peptide was tested individually, allowing every response to be assigned to a defined sequence. Longitudinal change was modelled using linear mixed-effects models, comparing total with median per-peptide responses to distinguish loss of specificities from loss of per-epitope strength. Analyses were stratified by booster type and vaccination strategy (homologous vs. heterologous).

**Results:**

Time strongly affected total S-specific IFN-γ responses (F(3, 96.6) = 33.16, p = 8 × 10^−15^) but not median per-peptide responses (F(3, 95.8) = 0.62, p = 0.606), indicating that the decline reflected contraction in the number of epitopes recognized rather than weakening of individual responses; the same dissociation held within both booster groups. Ad26.COV2.S boosting was associated with broader S-specific epitope recognition and greater durability, with a subset of responses persisting through Month 9, whereas BNT162b2 recipients showed higher cumulative IFN-γ magnitude through Month 6 — a difference already present before boosting (p = 0.012). N-specific responses declined despite stable anti-nucleocapsid antibody, although two N-derived regions (YGANKDGIIW, VTPSGTWLTY) were recognized at every timepoint.

**Discussion/conclusions:**

Booster platform and vaccination strategy shaped the magnitude, breadth and durability of SARS-CoV-2-specific T-cell responses, with the longitudinal decline appearing to be driven predominantly by narrowing of epitope breadth among Month 3-reactive peptides rather than reduced per-epitope function. The persistently immunogenic S and N epitopes identified here warrant further evaluation as candidates for multi-epitope T-cell vaccine design.

## Introduction

Since its emergence in late 2019, severe acute respiratory syndrome coronavirus 2 (SARS-CoV-2), the causative agent of coronavirus disease 2019 (COVID-19), has caused substantial global morbidity and mortality (Zheng, 2020). By late 2020, multiple vaccines had been deployed, markedly reducing hospitalizations and deaths worldwide (Li et al., 2022; Faraji et al., 2024). However, the emergence of immune-evasive variants and waning vaccine- or infection-induced immunity has complicated sustained control of transmission (Krause et al., 2021; Roederer et al., 2024). Reinfections are increasingly observed, though generally with reduced severity (Lipsitch et al., 2021). Despite the end of the public health emergency, intermittent outbreaks persist, underscoring the need for durable protective immunity. Booster vaccination, including updated formulations, has therefore been implemented to sustain immune protection (Chemaitelly et al., 2023). Nonetheless, optimal booster strategies and their durability across immunological contexts remain incompletely defined. In parallel, ongoing coronavirus evolution within animal reservoirs maintains the risk of future spillover and resurgence.

SARS-CoV-2 belongs to the *Beta-coronavirus* genus (subgenus *Sarbecovirus*) within the *Coronaviridae* family (Gorbalenya et al., 2020). It as an enveloped, positive-sense, single-stranded, non-segmented RNA genome encapsulated by a nucleocapsid (N) protein. Its outer envelope contains three structural proteins: envelope (E), membrane (M), and spike (S) proteins (Jahirul Islam et al., 2023). The S protein, a trimeric class I fusion glycoprotein, mediates viral entry by binding its receptor-binding domain (RBD) to the human angiotensin-converting enzyme 2 (hACE2) receptors on epithelial cells. It is therefore the principal target of neutralizing antibodies (nAbs) aimed at blocking viral entry. Consequently, all currently deployed COVID-19 vaccines, including the mRNA-based BNT162b2 and the adenoviral-vectored Ad26.COV-2.S encodes the S protein alone.

Following infection or vaccination, innate and adaptive immune responses act in concert to limit viral replication and clear infection. Neutralizing antibodies against the spike constitute key correlates of protection; however, they decline substantially within months after immunization or infection (Goldberg et al., 2021; Levin et al., 2021). This waning can reduce protection against infection including mild and asymptomatic cases (Andrews et al., 2022; Sankaranarayanan et al., 2022; Tan et al., 2023). This challenge is compounded by spike mutational diversity, which facilitates immune escape and sustained viral transmission (Harvey et al., 2021; Sinha et al., 2023; Yu et al., 2023). In contrast, T-cell responses are more durable and broadly cross-reactive across variants (Lu and Yamasaki, 2022; Zhou et al., 2024). T cells recognize peptide epitopes presented by major histocompatibility complex (MHC) molecules, many of which are conserved across SARS-CoV-2 variants (Geers et al., 2021; Sankaranarayanan et al., 2022). These responses are critical for limiting disease severity after antibody waning.

Although all currently deployed COVID-19 vaccines focus exclusively on the S protein, protective T-cell responses are also directed against immunodominant epitopes in other SARS-CoV-2 structural proteins, most notably the N and M. These responses are primarily acquired through natural infection and contribute to broad cellular immune memory that extends beyond S-specific immunity. The N protein is of particular immunological interest due to its high sequence conservation, abundant expression during infection, and strong recognition by CD4^+^ and CD8^+^ T-cells, making it a promising target for cross-variant and pan-coronavirus vaccine strategies (Gao et al., 2021; Zhang et al., 2022).

Despite extensive research on SARS-CoV-2 vaccination, important gaps remain in understanding the magnitude, specificity, breadth, and durability of vaccine-induced T-cell responses following booster immunization. These gaps are particularly relevant in African populations, where extensive human leukocyte antigen (HLA) diversity may influence antigen presentation, epitope immunodominance, and the quality of cellular immune responses (Copley et al., 2021). Consequently, the determinants and longevity of vaccine-induced cellular immunity in genetically diverse populations remain incompletely characterized, particularly across different booster platforms and immunological backgrounds, including hybrid immunity. Although both CD4^+^ and CD8^+^ T cells contribute to antiviral protection, CD8^+^ T cells are critical mediators of immunity through their ability to recognize and eliminate infected cells, thereby limiting viral replication and disease progression (Li et al., 2021; van der Ploeg et al., 2022). Furthermore, CD8^+^ T-cell responses are typically directed against peptides presented by major histocompatibility complex (MHC) class I molecules and often target conserved viral regions that are less susceptible to antibody escape.

This study, therefore, evaluated the longevity and antigen specificity of SARS-CoV-2-specific IFN-γ T-cell responses to spike (S) and nucleocapsid (N) peptides in a Ghanaian adult cohort following homologous and heterologous booster vaccination with BNT162b2 or Ad26.COV2.S at least six months after completing their primary vaccination series. *In silico* MHC class I restriction prediction was performed to identify putative CD8^+^ T-cell epitopes, and N-specific responses were assessed against a background of prior natural infection, which we have previously documented serologically in all participants of this cohort (Partey et al., 2026). By integrating functional T-cell measurements with immunogenetic analyses, this study provides insights into the durability and antigenic breadth of booster-induced cellular immunity in a genetically diverse population and identifies conserved, putativeCD8^+^ T-cell targets relevant for next-generation SARS-CoV-2 vaccine design.

## Materials and methods

### Ethics statement

This study received approval from the Institutional Review Board of the Noguchi Memorial Institute for Medical Research (NMIMR-IRB, approval number CPN 010/22-23). All participants voluntarily agreed to participate, and each participant signed a written informed consent form prior to inclusion in the study. All experiments were conducted in compliance with the principles of the Belmont Report and of the Declaration of Helsinki.

### Study design and study area

This was a longitudinal study to assess the durability of post-booster SARS-CoV-2-specific T-cell immunity in a fully vaccinated adult cohort. Participants had previously completed a primary COVID-19 vaccination series with one of the following vaccines: AstraZeneca (ChAdOx1-S), Moderna (mRNA-1273), Pfizer-BioNTech (BNT162b2), or Janssen (Ad26.COV2.S).

Participants were drawn from a cohort described previously (Partey et al., 2026), in which baseline SARS-CoV-2 spike- and nucleocapsid-specific antibody responses were characterized before and after booster vaccination. Participants were recruited from the Legon community in Accra, located in the Greater Accra Region of Ghana. Eligible participants were healthy adults between the ages of 18 and 60 years with no known immunodeficiencies or chronic medical conditions at the time of recruitment, as confirmed by clinical screening and medical history. The inclusion criteria were: hemoglobin level >10 g/dL, not being pregnant or nursing (for female participants), completion of primary vaccination at least 6 months prior, and willingness to receive a booster dose of either the BNT162b2 mRNA vaccine or the Ad26.COV2.S vaccine. All participants provided venous blood collection at scheduled time points during the follow-up period.

Participants were stratified into two groups based on the booster vaccine received: BNT162b2 and Ad26.COV2.S Booster Groups. They were further classified by vaccination strategy. Vaccination strategy was defined by the sequence of vaccine platforms used for the primary series and the booster dose. Those who received vaccines designed on the same platform for both doses (e.g., mRNA–mRNA or viral vector–viral vector) were classified as having received a homologous booster, while those who received vaccines developed using different platforms (e.g., mRNA primary followed by a viral vector booster, or vice versa) were classified as heterologous.

### Data and sample collection and booster vaccination

Blood samples were collected from the participants at four longitudinal time points: on day zero (before receiving a booster vaccine) and then at 3, 6, and 9 months after booster vaccine administration. Participant demographic data, clinical data, vaccination history were collected at study baseline using a study-specific questionnaire. SARS-CoV-2 infection status during the study period was assessed using structured questionnaires administered to participants at each sampling time point between May 2023 and January 2024. The questionnaires captured self-reported COVID-19–related symptoms, whether participants had undergone SARS-CoV-2 diagnostic testing since the preceding visit, and the results of any such tests. In addition, SARS-CoV-2 nucleocapsid-specific antibodies were measured in all participants at each of the four sampling timepoints as stated above. All participants were nucleocapsid-seropositive at enrolment, indicating prior natural infection, and antibody levels remained stable throughout follow-up, indicating that no incident infection occurred during the study period. Participants were monitored for any immediate adverse reactions post-vaccination, and booster doses were recorded in each participant’s medical records. Participants were allocated to booster groups based on individual preference. Approximately 40 mL of venous whole blood was drawn from every participant at each time point. Blood was collected in sodium heparin-treated tubes (368484, BD Biosciences) and immediately transported to the laboratory for processing. Peripheral blood mononuclear cells (PBMCs) were isolated and stored in liquid nitrogen (-196 °C) until required for T-cell assays.

### PBMC isolation, storage and recovery

PBMCs were isolated from fresh human whole blood by Ficoll-Paque density gradient centrifugation. Whole blood was diluted 1:1 with RPMI-1640 (R5886, Sigma) supplemented with L-glutamine (25030-024, Gibco) and penicillin-streptomycin (P0781, Sigma) and layered (≤25 mL per tube) on a Ficoll gradient. The tubes were centrifuged at 1200 × g for 20 minutes at room temperature with the brake disengaged. The PBMC layer was collected using a sterile serological pipette, pooled for each participant, and transferred to fresh centrifuge tubes. Cells were washed twice with RPMI-1640 containing 5% fetal bovine serum (R5) (120K20, Avantor) by centrifugation at 300 × g for 10 minutes at room temperature with brakes engaged. Washed cells were resuspended in R5 for counting and viability assessment using a Countess II automated cell counter (C10285, Life Technologies) and Trypan Blue exclusion. For cryopreservation, PBMCs were resuspended in freezing medium (90% FBS with 10% dimethyl sulfoxide) at a concentration of either 10 or 20 × 10^6^cells/mL. Aliquots of 1 mL were transferred into pre-chilled cryovials, frozen overnight at – 80 °C in a Mr. Frosty™ controlled-rate cooling container (5100-0001, Nalgene) and moved to liquid nitrogen (–196 °C) for long-term storage the next day.

When required for experiments, cryopreserved PBMCs were retrieved from liquid nitrogen storage and rapidly thawed in a 37 °C water bath for approximately 60 seconds, until a small ice fragment remained. Thawed cells were transferred to a pre-labelled 50 mL centrifuge tube and 20 mL of R5 medium added dropwise with gentle swirling to ensure even mixing and to minimize thermal shock. The cells were washed by centrifugation at 300 × g for 10 minutes at room temperature. After a second wash under similar conditions, the cell viability was assessed using the Trypan Blue exclusion method. Samples with viability ≥80% were deemed suitable for downstream experiments; those falling below this threshold were excluded from further analysis. Resuspended cells were rested overnight (16–20 hours) at 37 °C in a humidified incubator with 5% CO_2_, in tubes placed at a ~45° angle with loosened caps to allow gas exchange.

### SARS-CoV-2 immunodominant T cell epitope prediction and peptide selection

The NetMHCpan-4.1 immunoinformatic tool (available at NetMHCpan 4.1 - DTU Health Tech - Bioinformatic Services), which applies artificial neural networks trained on binding affinity and eluted ligand data to predict peptide–MHC class I interactions, was used to predict overlapping 10-mer peptides from the SARS-CoV-2 S and N proteins based on their binding affinities to globally prevalent HLA class I supertypes. The full-length protein sequences, 1273 amino acids for S and 419 amino acids (aa) for N, were uploaded in the FASTA format. Prediction parameters were configured to identify 10-mer peptides with binding thresholds set at a rank of ≤0.5% for strong binders and ≤2% for weak binders.

The analysis focused on seven globally predominant HLA class I supertypes based on the classification by (Sidney et al., 2008), including A01, A02, A03, A24, B07, B40 and B58. Since participants were not typed for HLA, we selected and tested T-cell epitopes predicted to bind across these supertypes to maximize population coverage and immunogenetic relevance. For the N protein, only the top 15 peptides with a binding affinity (BA) rank ≤0.5% across the selected HLA supertypes were included, ensuring the selection of the strongest predicted binders and prioritizing the most promising candidates. For the S protein, peptides with an Eluted Ligand (EL) rank ≤0.5% were prioritized, as this score reflects a high likelihood of being naturally processed and presented by MHC class I molecules on infected cells.

Once the top-binding epitopes were identified, they were cross-referenced with the sequences of pre-synthesized SARS-CoV-2 S and N peptides, which had been synthesized based on the Wuhan reference strain (GenBank Accession number YP_009724390.1) by Mimotopes VIC, in Australia. These antigens were synthesized as overlapping 15-mer peptides with a 10-amino-acid offset, spanning the full length of the S and N proteins. This overlap ensured that the predicted 9–10-mer epitopes were embedded within longer 15-mers, maximizing the likelihood of capturing naturally processed immunogenic sequences. Each synthetic peptide sequence had a unique ID, to which the predicted epitopes and their characteristics were matched, as shown in **Table 1**. Synthetic N peptides were designated with the prefix np (N peptide), followed by a numeric identifier (e.g., np25). S glycoprotein peptides were designated with the prefix SGP (S glycoprotein peptide), followed by a numeric identifier (e.g., SGP163).

**Table 1:** Predicted 10mer peptides and corresponding synthetic 15mer peptide reference IDs.

Predicted peptide	EL_Rank (%)	HLA haplotype	15mer peptide sequence	15mer sequence ID
S Peptides				
VYYHKNNKSW	0.1995	HLA A24:02	HKNNKSWMESEFRVY*	SGP030
SSANNCTFEY	0.1587	HLA A01:01	EFRVYSSANNCTFEY	SGP032
YLQPRTFLLK	0.153	HLA A03:01	YVGYLQPRTFLLKYN	SGP054
ATRFASVYAW	0.2392	HLA B58:01	VFNATRFASVYAWNR	SGP069
KLNDLCFTNV	0.3539	HLA A02:01	GVSPTKLNDLCFTNV	SGP077
RQIAPGQTGK	0.0396	HLA A03:01	EVRQIAPGQTGKIAD	SGP082
KLPDDFTGCV	0.3198	HLA A02:01	YNYKLPDDFTGCVIA	SGP085
NLDSKVGGNY	0.1564	HLA A01:01	WNSNNLDSKVGGNYN	SGP088
NTSNQVAVLY	0.1315	HLA A01:01	GTNTSNQVAVLYQDV	SGP121
IHADQLTPTW	0.1029	HLA B58:01	PVAIHADQLTPTWRV	SGP125
TEILPVSMTK	0.1532	HLA A03:01	SVTTEILPVSMTKTS	SGP145
KQIYKTPPIK	0.0347	HLA A03:01	VFAQVKQIYKTPPIK	SGP157
ILPDPSKPSK	0.2694	HLA A03:01	NFSQILPDPSKPSKR	SGP161
KRSFIEDLLF	0.2545	HLA B58:01	KPSKRSFIEDLLFNK	SGP163
LADAGFIKQY	0.0798	HLA A01:01	VTLADAGFIKQYGDC	SGP166
LLTDEMIAQY	0.0225	HLA A01:01	LPPLLTDEMIAQYTS	SGP173
VEAEVQIDRL	0.1392	HLA B40:01	KVEAEVQIDRLITGR	SGP198
HVTYVPAQEK	0.1401	HLA A03:01	VFLHVTYVPAQEKNF	SGP213
SLIDLQELGK	0.2339	HLA A03:01	KNLNESLIDLQELGK	SGP239
LIDLQELGKY	0.1049	HLA A01:01	SLIDLQELGKYEQYI	SGP240
FDEDDSEPVL	0.2454	HLA B40:01	GSCCKFDEDDSEPVL	SGP251
SEPVLKGVKL	0.2057	HLA B07:02	FDEDDSEPVLKGVKL	SGP252
N peptides				
KFPRGQGVPI	0.0122	HLA B07:02	KEDLKFPRGQGVPIN	np13
SSPDDQIGYY	0.0184	HLA A01:01	TNSSPDDQIGYYRRA	np16
LLNKHIDAYK	0.0565	HLA A03:01	ILLNKHIDAYKTFPP	np71
KTFPPTEPKK	0.0571	HLA A03:01	HIDAYKTFPPTEPKK	np72
VTPSGTWLTY	0.1242	HLA A01:01	GMEVTPSGTWLTYTG	np65
QFAPSASAFF	0.2155	HLA A24:02	QFAPSASAFFGMSRI	np62
YGANKDGIIW	0.2166	HLA B58:01	LPYGANKDGIIWVAT	np25
FPRGQGVPIN	0.2711	HLA B07:02	FPRGQGVPINTNSSP	np14
DLSPRWYFYY	0.305	HLA A01:01	MKDLSPRWYFYYLGT	np21
GTGPEAGLPY	0.3083	HLA A01:01	YYLGTGPEAGLPYGA	np23
QLPQGTTLPK	0.3814	HLA A03:01	AIVLQLPQGTTLPKG	np32
KKPRQKRTAT	0.4011	HLA B07:02	AAEASKKPRQKRTAT	np51
FGMSRIGMEV	0.4114	HLA A02:01	ASAFFGMSRIGMEVT	np63
RPQGLPNNTA	0.439	HLA B07:02	RSKQRRPQGLPNNTA	np08
RNPANNAAIV	0.4891	HLA B07:02	IGTRNPANNAAIVLQ	np30

Immunodominant peptides predicted using NetMHCpan-4.1. Predictions were performed using default settings, with peptides selected based on strong binding affinity (rank ≤ 0.5%) across seven globally prevalent HLA class I supertypes. Sequence ID denotes the synthesized 15mer peptides that contain the 9-10mer predicted epitopes (in red).

*For this epitope, only a partial sequence of the 10mer epitopes was contained within the 15mer sequence.

SnapGene Viewer (version 8.0.2) was used to map the sequences of synthetic peptide IDs to their corresponding regions within the full-length S and N proteins. The mapped regions for the S and N proteins, which also show the amino acid positions within the protein primary sequences, are presented in **Figures 1** and **2**.

**Figure 1:**
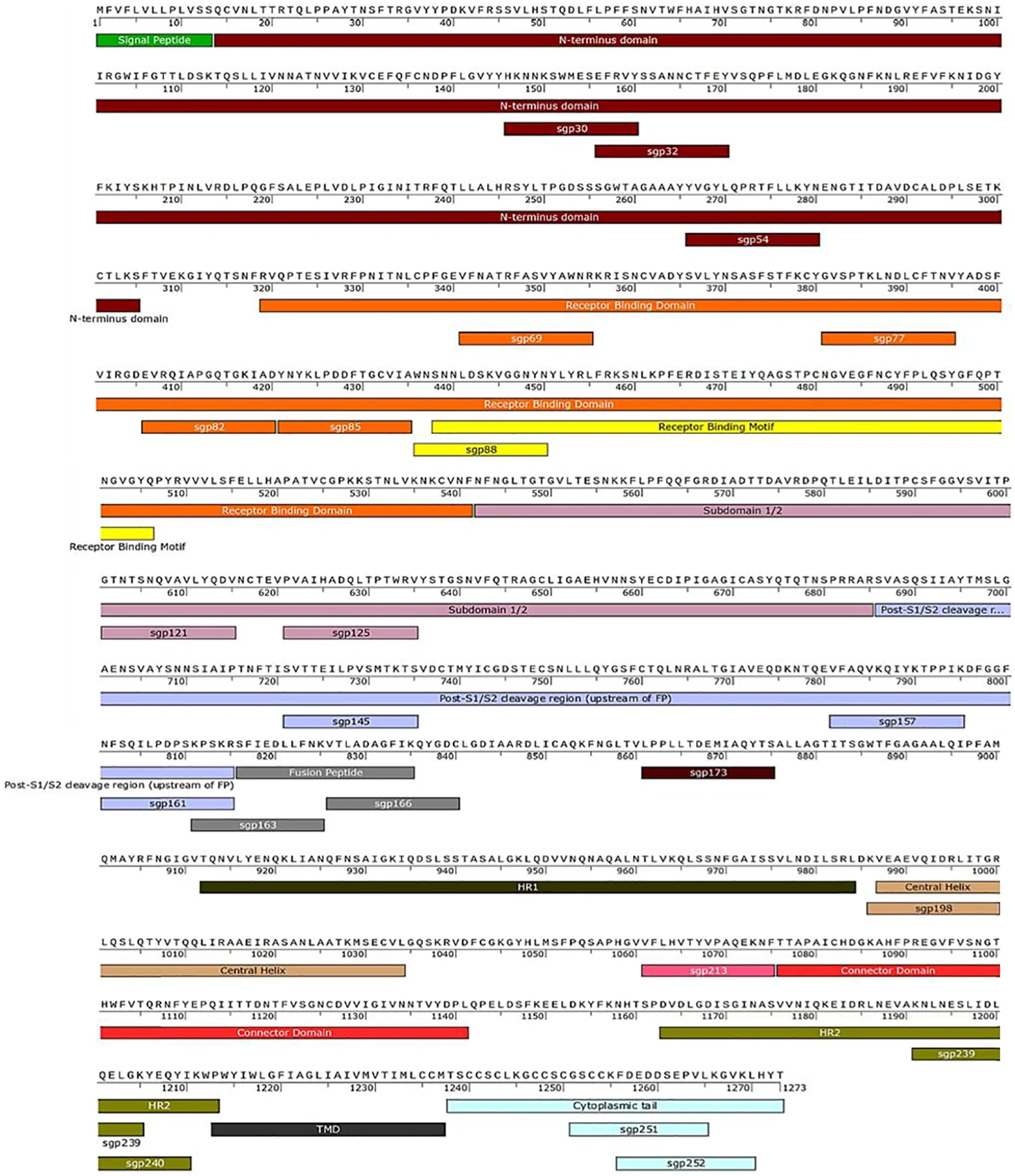
Architecture of the S protein (1273aa) and map of 22 predicted S glycoprotein (SGP) peptides. The amino acid sequence of the S protein with demarcation of the Signal peptide, NTD, RBD, RBM, Subdomain 1/2, Post-S1/S2 cleavage region, S1/S2 cleavage site, the Fusion peptide, Heptad repeats 1 and 2, Central Helix, Connector domain, HR2, TMD and the Cytoplasmic tail. The signal peptide is colored dark lime green. The other colored regions are: Maroon = NTD (SGP30 – SGP54); Orange = RBD (SGP69 – SGP85); Yellow = RBM (SGP88), Pinkish lavender = Subdomain 1/2 (SGP121 and SGP125); Cyan = S1/S2 cleavage site; Light blue = Post S1/S2 cleavage region (SGP145, SGP161 and SGP157); Gray = Fusion peptide (SGP163 and SGP166); Olive tone = HR1; Sandy tan = Central Helix; Red = Connector domain; Olive = HR2 (SGP239 and SGP240); Charcoal Gray = TMD; Light Cyan= Cytoplasmic tail. This image was obtained from the SnapGene Viewer bioinformatics tool (version 8.0.2).

**Figure 2:**
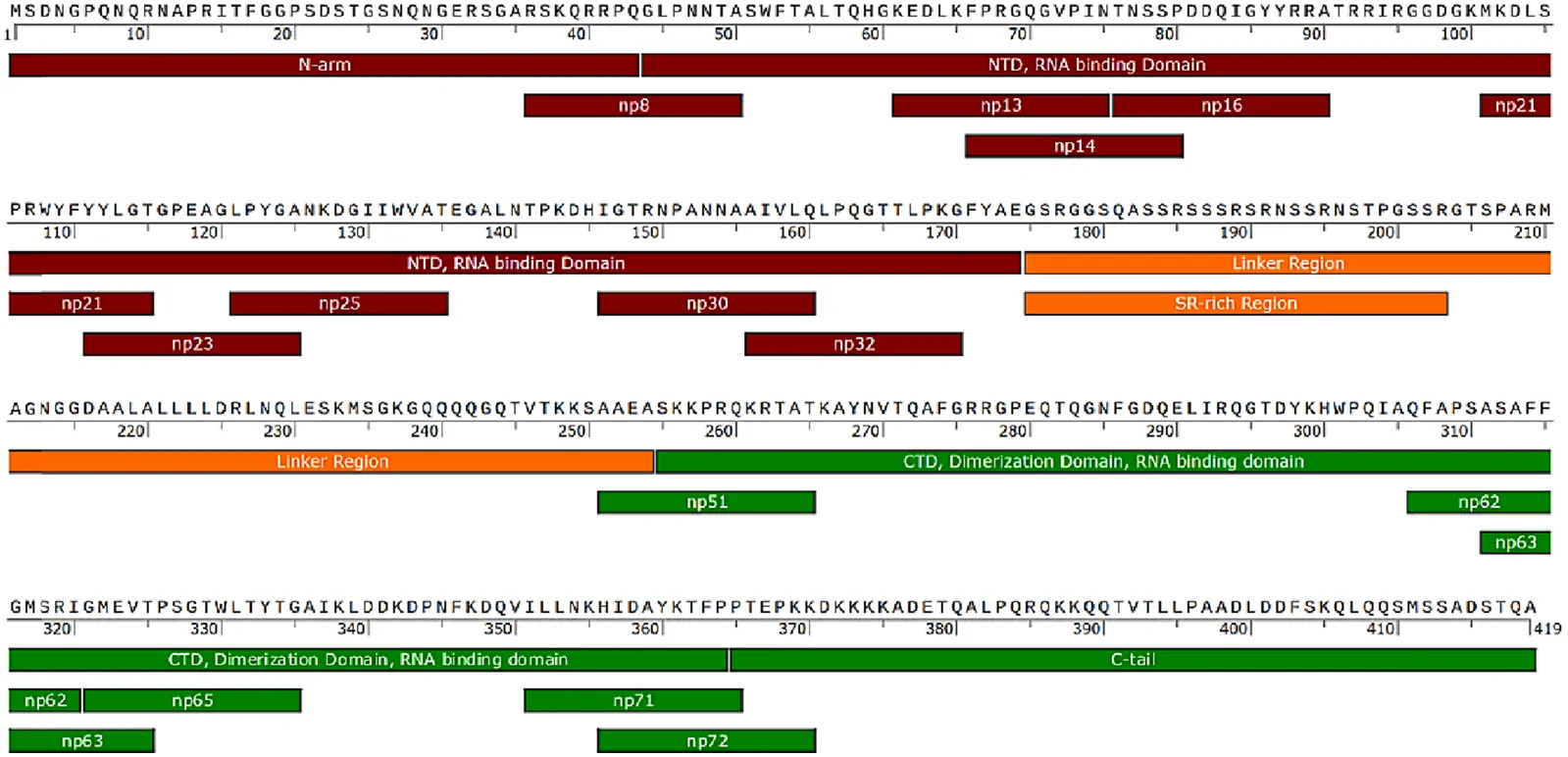
Architecture of the N protein (419aa) and map of 15 predicted N peptides (np). The amino acid sequence of the N protein with demarcation of the N-, Linker- and C-termini. The N-terminus is colored maroon, the Linker in orange and the C-terminus in green. Maroon = N-arm (np 1 –np5), NTD (np 8 – np 32); Orange = Linker region; Green = CTD (np 51–np 72). This image was obtained from the SnapGene Viewer bioinformatics tool (version 8.0.2).

All peptides were synthetically produced by a commercial peptide synthesis service (Mimotopes, VIC, Australia) using solid-phase peptide synthesis (SPPS) to a purity of 90% or greater and tested for LPS/endotoxins. However, independent testing for LPS/endotoxins was not performed prior to running experiments. The lyophilized peptides were initially dissolved in 40 μL of dimethylsulfoxide (DMSO) for an hour, and further diluted by adding 4,960 μL of phosphate-buffered saline, pH 7, to a stock concentration of 1 mg/ml. Prior to addition to ELISpot plates, peptides were further diluted to a concentration of 20 µg/mL in blocking buffer consisting of 10% cell culture-tested Fetal Bovine Serum (FBS, Gibco, ref number: A56708-01) in RPMI-1640, supplemented with 1% penicillin-streptomycin, sodium pyruvate, L-glutamine and Hepes.

### Ex vivo IFN-γ ELISpot assay

The *ex vivo* IFN-γ enzyme-linked immunospot (ELISpot) assay was performed according to our standard laboratory protocol as previously described (Kusi et al., 2020; Kusi et al., 2022). Briefly, participant PBMCs were thawed and rested for up to 18 hours before they were stimulated with 10 μg/mL of the selected immunodominant S and N peptides in pre-coated ELISpot plates (Mabtech, product number 3420-4APT-10). The medium used for cell culture was 10% cell culture tested FBS in RPMI-1640 with 1% Penstrep, Sodium Pyruvate, L-glutamine and Hepes. Concanavalin A (Con A, Sigma Aldrich, USA) (1.25 µg/mL) was used as a positive control, and PBMCs incubated with culture medium only were used as negative controls. PBMCs were added at a concentration of 200,000 cells per well for test and negative control conditions and for positive control wells, 20,000 cells per well were stimulated with Con A-. All the samples were plated in duplicate and incubated at 37 °C in a humidified incubator with 5% CO_2_ for 24 hours. After substrate incubation and plate development, the number of IFN-γ-producing cells was estimated using an automated ELISpot plate reader (AID GmbH, Germany) and the data were exported into Microsoft Excel for analysis. Any assays in which the negative control exceeded 10 spot-forming cells per million (sfc/m) were considered invalid, following the definition established in the studies referenced above. Any plates failing to meet these criteria were flagged for re-running with cryopreserved PBMCs from the same participant.

Cryopreserved PBMCs from 46 participants were tested in the *ex-vivo* ELISpot IFN-γ assay against predicted S-derived peptides. Additionally, a subset of 16 participants was tested against the predicted N-derived peptides. N antigen testing was limited to samples with enough remaining PBMCs after S peptide analysis. At baseline (Month 0) and Month 3, PBMCs were stimulated with the full panel of predicted peptides. At Months 6 and 9, peptide stimulation was restricted to peptides that had demonstrated positivity at Month 3, such that later timepoints reflect longitudinal persistence of the previously detected peptide-specific responses. This approach enhanced assay efficiency by restricting the longitudinal monitoring of immunodominant peptides with demonstrated post-boost responsiveness. It also reflects the immunological principle of prioritizing epitopes that drive robust *in vivo* T-cell activation, responses more likely to contribute to durable and protective immunity (Grifoni et al., 2020; Grifoni et al., 2021; Binayke et al., 2024). However, we acknowledge that this approach may exclude delayed responses that may not be evident at baseline or at Month 3 and could be detectable by Months 6 and/or 9.

### Data and statistical analysis

Descriptive comparisons of demographic characteristics were conducted using independent-sample t-tests or chi-square (χ²) tests, as appropriate. Peptide responses were classified as positive or negative based on predefined criteria: a peptide-stimulated well was considered positive if (Zheng, 2020) the spot-forming cells per million (sfc/m) PBMCs at least doubled (≥ 2) relative to control wells and (Li et al., 2022) the difference in spot count between test and control wells was at least 10. This definition was adapted from and validated in previous studies (Kusi et al., 2020; Kusi et al., 2022; Belmonte et al., 2023). Raw spot count data were processed using deterministic spreadsheet-based workflows to apply the positivity criteria. Group-level comparisons of total peptide positivity (e.g., homologous vs. heterologous) were conducted using chi-square or Fisher’s Exact Tests, depending on expected cell counts. These analyses involved non-overlapping single-hypothesis tests and therefore did not require correction for multiple comparisons due to the limited number of pre-specified analyses. However, we acknowledge that repeated testing may increase the risk of Type I error.

For each participant, IFN-γ spot counts were aggregated across all tested peptides that elicited a detectable response at the respective timepoints, and values were log_10_-transformed prior to analysis. The median responses between groups at specific time points were compared using the Wilcoxon rank-sum test. To account for within-participant correlation arising from repeated measurements and unbalanced observation numbers across timepoints, longitudinal changes in IFN-γ magnitude were modelled using linear mixed-effects models with participant identity included as a random intercept. Timepoint was treated as a fixed effect. Statistical inference therefore reflects within-participant changes in the magnitude of antigen-specific IFN-γ responses over time. For each fitted model, estimated marginal means, Tukey-adjusted pairwise contrasts with effect sizes, and omnibus F-tests were extracted using a consistent analytical framework. All statistical analyses were performed in R (version 2025.05.0 + 496), with statistical significance defined as p < 0.05.

## Results

### Demographic characteristics of the study population

This study included 46 participants, comprising 27 males (56.7%) and 19 females (41.3%), and there was no difference in their distribution between the two booster vaccination groups (**Table 2**, *p* = 0.24). The mean age was 25.48 ± 7.34 years and was not significantly different between the vaccination groups (*p* = 0.69). Each booster group included 23 participants. In the BNT162b2-BioNTech group, 43.5% (*n* = 10) received homologous mRNA boosting and 56.5% (*n* = 13) received a heterologous boost following a viral vector primary series. In contrast, 69.6% (*n* = 16) of the Ad26.COV2.S group received homologous boosting, while 30.4% (*n* = 7) received a heterologous booster following primary mRNA vaccination, and these proportions were not significantly different between the booster vaccination groups (**Table 2**, *p*= 0.38). A total of 29 participants (63.0%) completed their primary vaccination with a viral vector vaccine (AstraZeneca or Ad26.COV2.S), while 17 (37.0%) received mRNA primary vaccination, but this difference was not statistically significant (*p* = 0.077).

**Table 2:** Demographic and baseline clinical characteristics of study participants, stratified by study group.

Characteristic	Category	Total (n = 46)	Ad26.COV2.S (n = 23)	BNT162b2 (n = 23)	p-value
**Sex**	Male	27 (56.7%)	14 (60.9%)	13 (56.5%)	0.24
	Female	19 (41.3%)	9 (39.1%)	10 (43.5%)	
**Age (years)**	Mean ± SD	25.48 ± 7.34	25.04 ± 6.51	25.91 ± 7.88	0.69
**Vaccination regimen**	Homologous	26 (56.5%)	16 (69.6%)	10 (43.5%)	0.38
	Heterologous	20 (43.5%)	7 (30.4%)	13 (56.5%)	
**Primary vaccination**	Viral-vectored vaccine	29 (63.0%)	16 (43.5%)	13 (47.8%)	0.077
	mRNA vaccine	17 (37.0%)	7 (26.1%)	10 (8.7%)	

Categorical variables (Sex, Vaccination Regimen, and Primary Vaccination) are presented as counts and percentages [n (%)], with p-values calculated using Chi-square tests to assess differences in proportions between groups. Age is reported as mean ± standard deviation, with p-values derived from Welch’s two-sample t-test to account for potential differences in group variances.

### Temporal dynamics of S-specific T-cell responses

At baseline (month 0), 22 of the 46 participants exhibited positive IFN-γ responses to at least one of the 22 S peptides tested (**Supplementary Table 1**). In all, a total of 81 positive peptide responses were recorded, with 35 in the Ad26.COV2.S group and 46 in the BNT162b2 group (**Table 3**; **Supplementary Table 1**). Each peptide elicited at least one positive response. The most frequently recognized epitopes were in the fusion peptide region, peptides LADAGFIKQY (aa 826–840, *n* = 8) and KRSFIEDLLF (aa 811–825, *n* = 6) and the NTD peptide SSANNCTFEY (aa 156–170, *n* = 6) (**Supplementary Table 1**). Two participants (P025 and P056) exhibited the highest breadth of response, recognizing 12 peptides each (**Table 3**). Median IFN-γ levels were significantly higher in the BNT162b2 group than in the Ad26.COV2.S group (*p* = 0.012, **Figure 3A**).

**Table 3:** Vaccination strategy and number of positive S peptides per participant.

Participant ID	Vaccination strategy	S peptide positivity per participant at:			
		Month 0	Month 3	Month 6	Month 9
Ad26.COV2.S (viral vector)-boosted participants					
J006	Heterologous	0	1	1	0
J023	Homologous	11	0	N/A	N/A
J028	Homologous	3	7	1	1
J031	Homologous	3	2	2	0
J032	Homologous	0	0	N/A	N/A
J034	Homologous	0	0	N/A	N/A
J035	Homologous	0	18	1	0
J039	Homologous	0	0	N/A	N/A
J040	Homologous	0	0	N/A	N/A
J059	Homologous	4	17	0	0
J060*	Homologous	8	N/A	N/A	N/A
J076	Homologous	1	0	N/A	N/A
J113	Heterologous	0	2	0	0
J114	Heterologous	0	6	2	0
J125	Heterologous	0	0	N/A	N/A
J166	Heterologous	0	0	N/A	N/A
J171	Homologous	0	8	5	8
J172*	Homologous	1	N/A	N/A	N/A
J176	Heterologous	0	2	0	0
J180	Heterologous	2	2	0	0
J183	Homologous	0	3	0	0
J189	Homologous	1	1	0	0
J206	Homologous	1	1	1	0
BNT162b2 (mRNA)-boosted participants					
P005	Heterologous	1	0	N/A	N/A
P008	Heterologous	0	0	N/A	N/A
P011	Heterologous	0	3	0	0
P012	Heterologous	1	0	N/A	N/A
P015	Homologous	6	1	0	0
P018	Heterologous	0	5	0	0
P023	Homologous	1	0	N/A	N/A
P025	Heterologous	12	0	N/A	N/A
P030	Heterologous	1	0	N/A	N/A
P032	Heterologous	0	6	6	0
P040	Heterologous	1	0	N/A	N/A
P051	Homologous	3	4	4	0
P056	Heterologous	12	2	0	0
P059	Homologous	0	2	0	0
P060	Heterologous	0	1	0	1
P064	Heterologous	0	1	0	0
P066	Homologous	0	1	0	0
P079	Homologous	0	0	N/A	N/A
P084	Heterologous	2	2	0	0
P085	Homologous	0	1	0	0
P089	Homologous	5	3	0	0
P099	Homologous	1	0	N/A	N/A
P109	Homologous	0	2	0	0
Total positive peptides at each time point		81	104	23	10

Participants J060* and J172* were excluded from the Month 3 analysis due to poor PBMC viability. Homologous vaccinations are indicated in blue. N/A indicates unavailable values.

**Figure 3:**
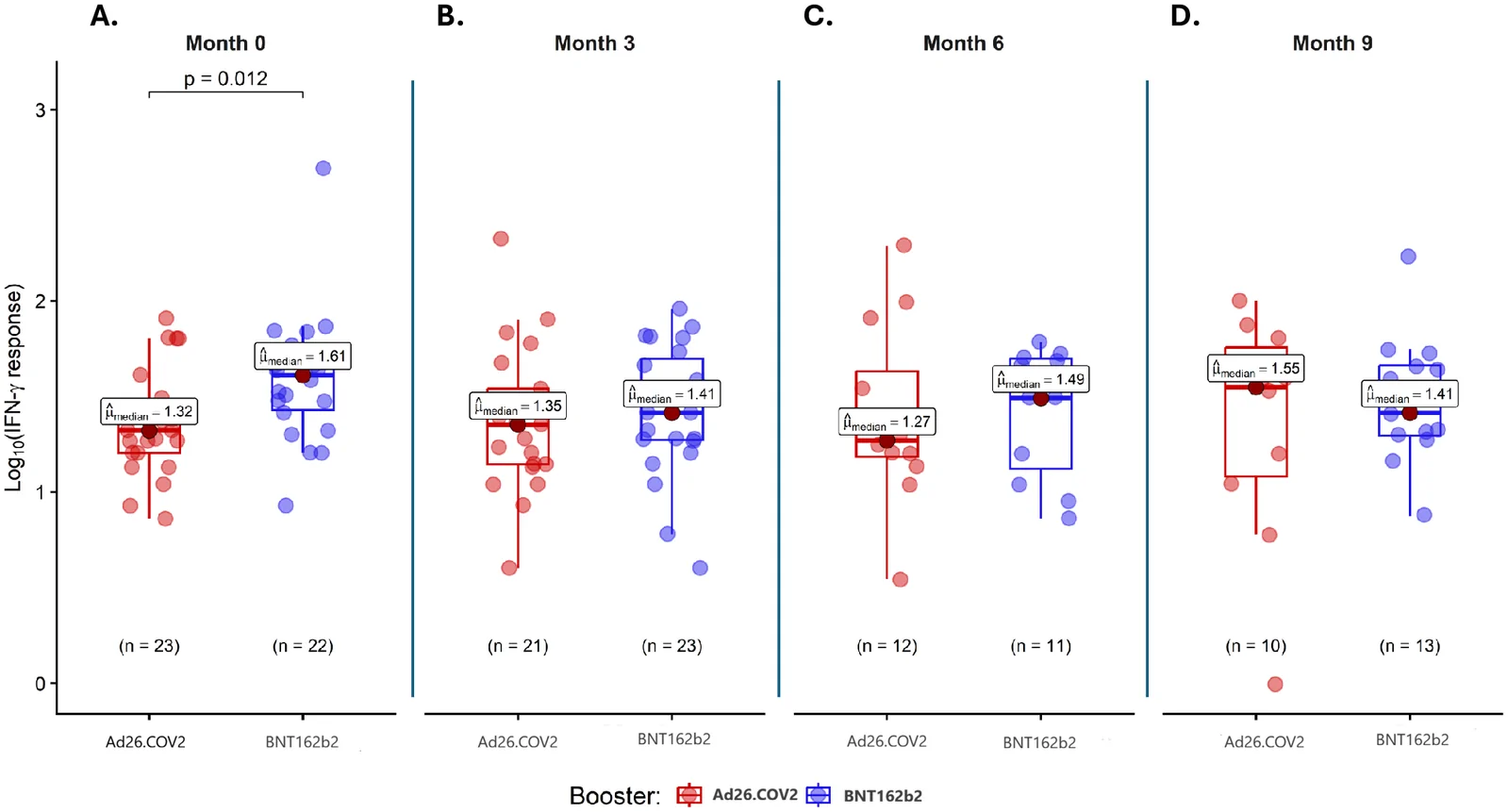
Comparison of S-specific T-cell IFN-γ activity between Ad26.COV2.S and BNT162b2 booster-vaccinated groups at time points **(A)** Month 0 (pre-booster), **(B)** Month 3, **(C)** Month 6, and **(D)** Month 9 following booster vaccination. Boxplots display the distribution of log_10_-transformed IFN-γ spot counts in response to stimulation with predicted SARS-CoV-2 S peptides. Each data point represents an individual participant’s summed peptide responses at the specified time point. Analyses at Months 6 and 9 are restricted to participants with persisting responses from Month 3. Median IFN-γ levels are indicated by dark red dots and labelled at each time point; box edges represent the interquartile range (IQR), and whiskers extend to 1.5 × the IQR. Sample sizes (n) are shown below each group. Only statistically significant p-values from Wilcoxon rank-sum tests at each time point are displayed above the corresponding brackets. Red and blue represent the Ad26.COV2.S and BNT162b2 booster groups, respectively.

In Month 3, positive peptide responses were observed in 27 of the 46 participants, and this included 11 participants who also had baseline responses (**Table 3**). Thus, 18 participants who had no positive peptide responses at Month 0 made positive peptide responses at Month 3. The breadth of peptide recognition, defined as the number of distinct peptides eliciting a response, was higher in the Ad26.COV2.S group, which had 70 peptide-positive responses compared to 34 in the BNT162b2 group (p = 4.4 × 10^−5^). The most recognized peptides at this time point were in the RBD (KLPDDFTGCV, aa 421–435, *n* = 8; KLNDLCFTNV, aa 381–395, *n* = 7), Subdomain 1/2 (IHADQLTPTW aa 621–635, *n* = 8), and NTD (SSANNCTFEY, aa 156–170, *n* = 7) regions. Participants J035 (*n* = 18) and J059 (*n* = 17) exhibited the broadest individual responses at this time point (**Table 3**). BNT162b2-boosted participants showed comparatively higher, though insignificant, IFN-γ levels (*p* = 0.39, **Figure 3B**).

At Month 6, peptide recognition declined to 23 total responses (**Supplementary Table 1**), comprising 13 in the Ad26.COV2.S booster group and 10 in the BNT162b2 booster group (**Table 3**). Despite this reduction in response frequency, IFN-γ levels between the groups remained significantly unchanged (*p* = 0.9, **Figure 3C**). By Month 9, IFN-γ levels remained comparable (*p* = 0.80, **Figure 3D**), with only 10 peptide-specific responses detected overall, nine in the Ad26.COV2.S booster group and one in the BNT162b2 booster group. Participant J171 accounted for 8 of these responses (**Table 3**).

From Months 0 to 6, the BNT162b2 group showed consistently higher IFN-γ activity than the Ad26.COV2.S group, with a statistically significant difference at baseline (**Figure 3**). By Month 9, IFN-γ levels were higher in the Ad26.COV2.S booster group, although the difference was statistically insignificant. Of the 22 S peptides tested at Months 0 and 3, 19 elicited responses through Month 6, and nine remained immunoreactive at Month 9 (**Supplementary Table 1**). Notably, participant J028 recognized at least one S epitope at every time point, whereas participant J171 had significant peptide responses at Months 3, 6, and 9 following Ad26.COV2.S booster vaccination (**Table 3**). Overall, there was marked inter-individual heterogeneity in the magnitude and longitudinal trajectory of IFN-γ responses among participants responding to the same peptide. While some participants maintained relatively stable responses across the four sampling time points, others exhibited increasing, decreasing, or fluctuating response patterns, highlighting considerable variability in vaccine-induced cellular immunity. (**Supplementary Figure 1**).

To distinguish between changes in overall participant response magnitude and changes in per-peptide response strength, we compared mixed-effects models using total (summed across peptides) versus median IFN-γ responses at the participant level. Across the entire booster-vaccinated cohort, S-specific IFN-γ responses remained stable three months following booster vaccination (p = 0.976; **Figure 4**; **Supplementary Excel File 1**), indicating maintenance of early post-booster responses. This was followed by a pronounced decline by Month 6 and subsequent stabilization through Month 9.

**Figure 4:**
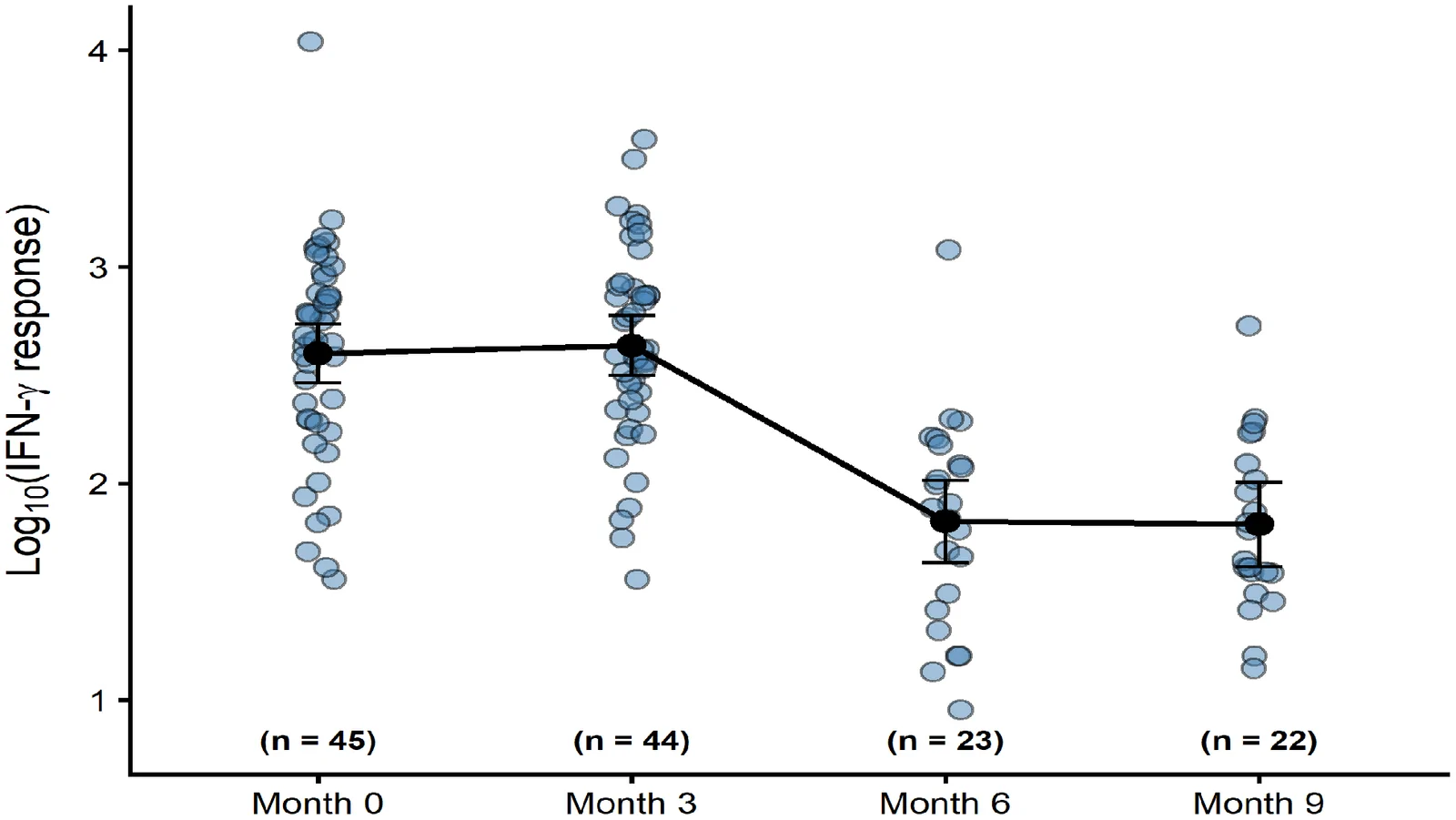
Longitudinal trend of S-specific T cell IFN-γ response in the entire vaccinated cohort. Participant-level IFN-γ responses (log_10_-transformed) are shown at Months 0 (pre-booster), 3, 6, and 9 post-booster. The S-specific IFN-γ response at each time point represents the sum of the IFN-γ responses to all previously assessed S-peptide-specific reactions. Light blue dots represent individual participants with detectable responses at each time point. Black dots indicate marginal group means estimated from a linear mixed-effects model with participant as a random intercept. Error bars indicate 95% confidence intervals, and black lines connect group means to visualize temporal trends. Numbers below each time point indicate the number of participants contributing data (n). Analyses are restricted to persisting responses (total IFN-γ > 0), such that the absence of a response is not treated as zero. Estimated mean values at each time point are listed in the **Supplementary Excel File**.

Significant reductions in IFN-γ levels were observed between Month 0 and Months 6 and 9, as well as between Month 3 and Months 6 and 9, all with large standardized effect sizes (d ≈ 1.8–2.0; **Supplementary Excel File 2**), indicating marked contraction of cumulative S-specific T-cell responses over time. No significant reduction in IFN-γ levels was detected between Month 6 and Month 9 (p = 1.00), consistent with stabilization of the remaining S-specific T-cell response beyond six months post-boost. Sensitivity analyses confirmed a strong overall effect of time on total IFN-γ levels (F (3, 96.6) = 33.16, p = 8 × 10^−15^), reflecting a decline in cumulative S-specific responses across time points. In contrast, median per-peptide IFN-γ responses did not change significantly over time (F(3, 95.8) = 0.62, p = 0.606; **Supplementary Excel File 3**), indicating preservation of per-epitope functional strength.

We repeated these analyses for each of the two booster vaccine types separately. In the Ad26.COV2.S group, S-specific IFN-γ responses appeared to increase between Month 0 and Month 3, although there was no significant change in model-estimated marginal means or pairwise contrasts was detected (**Figure 5A**, **Supplementary Excel File 2**). Beyond Month 3, a significant decline in cumulative S-specific IFN-γ output was observed, with reductions evident between Month 0 and Months 6 and 9, as well as between Month 3 and later time points. These declines were accompanied by medium-to-large standardized effect sizes, while no significant difference was detected between Months 6 and 9, indicating stabilization of persisting responses at later time points. Sensitivity analyses demonstrated a significant longitudinal effect of time on total IFN-γ responses (F(3, 50.5) = 7.48, p = 3.1 × 10^−4^), whereas median per-peptide responses did not show similar significance (F (3, 61) = 0.29, p = 0.835), suggesting that longitudinal changes were driven primarily by loss of responding peptides (i.e., reduced epitope breadth) rather than weakening of individual peptide responses (**Supplementary Excel File 3**).

**Figure 5:**
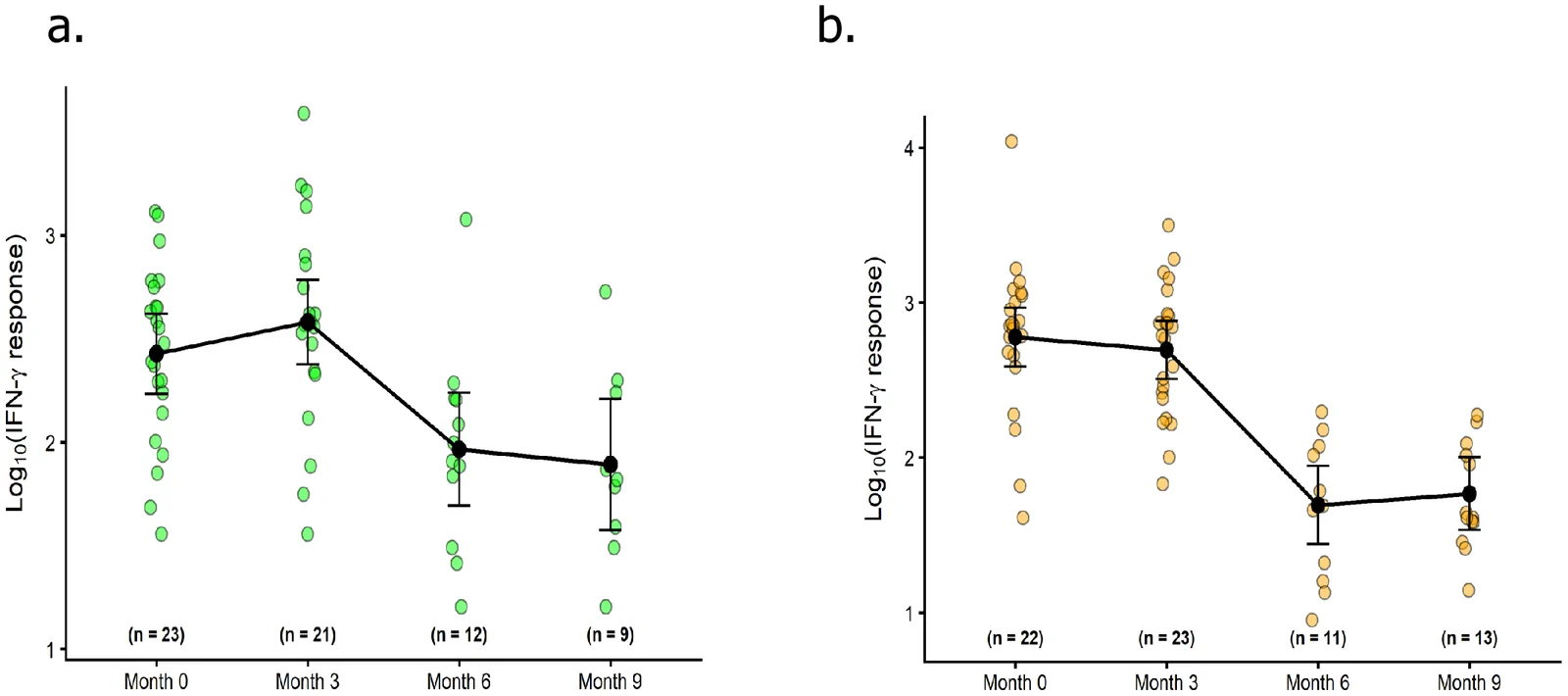
Longitudinal trend of S-specific T Cell IFN-γ response among **(a)** Ad26.COV2.S and **(b)** BNT162b2 booster recipients. Participant-level IFN-γ responses (log_10_-transformed) are shown at Months 0 (pre-booster), 3, 6, and 9 post-booster. The S-specific IFN-γ response at each time point represents the sum of the IFN-γ responses to all previously assessed S-peptide-specific reactions. Green (Ad26.COV2.S) and orange (BNT162b2) dots represent individual participants with detectable responses at each time point. Black dots indicate marginal group means estimated from a linear mixed-effects model with participant as a random intercept. Error bars indicate 95% confidence intervals, and black lines connect group means to visualize temporal trends. Numbers below each time point indicate the number of participants contributing data (n). Analyses are restricted to persisting responses, such that the absence of a response is not treated as zero. Estimated mean values at each time point are listed in the **Supplementary Excel File**.

For the BNT162b2 group, pairwise contrasts of model-estimated marginal means revealed no significant change in cumulative S-specific IFN-γ magnitude between Month 0 and Month 3 (**Figure 5B**, p = 0.847, **Supplementary Excel File 2**). In contrast, pronounced and highly significant reductions were observed between Month 0 and Months 6 and 9, as well as between Month 3 and Months 6 and 9, with very large standardized effect sizes, indicating marked contraction of S-specific T-cell responses over time. No significant difference was detected between Months 6 and 9 (p = 0.954), suggesting stabilization of the residual response after six months. Sensitivity modelling confirmed a strong overall effect of time on total IFN-γ output (F (3, 45.2) = 38.51, p = 1.65 × 10^−^¹²), whereas median per-peptide IFN-γ responses did not change significantly over time (F(3, 44.8) = 1.55, p = 0.215) (**Supplementary Excel File 3**). Collectively, these findings indicate that the observed longitudinal decline in S-specific IFN-γ responses following BNT162b2 boosting is driven predominantly by a reduction in the breadth of responding peptides rather than attenuation of per-peptide response strength.

### Evaluation of homologous versus heterologous vaccination strategies

In the Ad26.COV2.S booster group, two participants (J028 and J171) in the homologous subgroup (those who received Ad26.COV2.S viral vector booster following viral-vector primary series; *n* = 16) exhibited positive responses to at least one S peptide at all post-booster timepoints (Months 3, 6, and 9; **Table 3**). Three participants (J031, J035, J206) responded positively at Months 3 and 6, while another three (J059, J183, J189) made post-booster positive responses only at Month 3.

In the heterologous subgroup (mRNA-based primary vaccination followed by Ad26.COV2.S- booster; *n* = 7), two participants (J006, J114) showed positive post-booster responses at Months 3 and 6, and three (J113, J176, J180) responded only at Month 3. Only one member (J180) had a positive response at baseline (Month 0), and none showed S peptide recognition at Month 9 (**Table 3**). In contrast, epitope recognition frequencies remained higher in the homologous subgroup at months 0 (*p* = 0.007), 3 (*p* = 0.0001), and 9 (*p* = 0.035) (**Supplementary Table 2**). Subgroup comparisons at individual time points showed significantly higher IFN-γ levels in the homologous booster cohort at Month 0 (*p* = 0.027; **Supplementary Figure 2**), with no statistically significant differences observed between the cohorts at subsequent time points.

In the BNT162b2 booster group, none of the participants in the homologous subgroup (mRNA-based vaccination for both the primary series and booster; *n* = 10) demonstrated sustained positive responses to S peptides across all post-booster time points (**Table 3**). One individual (P051) recognized at least three S peptides from Month 0 to Month 6, while six others (P015, P059, P066, P085, P089, P109) exhibited positive responses at Month 3 only. No homologous-boosted participants showed S peptide recognition at Month 9.

Similarly, in the BNT162b2 heterologous subgroup (mRNA booster following viral vector primary vaccination; *n* = 13), no participant exhibited persistent responses across all post-booster time points. One participant (P060) responded at months 3 and 9, another (P032) at months 3 and 6, while five (P011, P018, P056, P064, and P084) responded only at month 3 (**Table 3**). Additionally, no significant differences in IFN-γ activity or epitope recognition frequency were observed between homologous and heterologous subgroups at any time point (**Supplementary Figure 3**; **Supplementary Table 2**).

### Temporal dynamics of N-specific T-cell responses

All participants were nucleocapsid-seropositive at baseline, indicating universal prior SARS-CoV-2 exposure before booster vaccination (Partey et al., 2026). Anti-nucleocapsid antibody levels remained stable across all four sampling timepoints, and no participant reported a laboratory-confirmed SARS-CoV-2 infection or a positive diagnostic test result during the nine-month follow-up period. Participants were not stratified by infection status for downstream analyses, as the cohort was uniformly prior-exposed and no infection-naive comparison group existed among our participants.

The COVID-19 vaccines administered to this cohort encode the S protein alone, whereas the N protein contains immunologically relevant epitopes acquired only through natural infection. Consequently, the detection of N-specific T-cell responses in vaccinated individuals is generally interpreted as evidence of prior or intercurrent natural exposure to SARS-CoV-2, although the timing and clinical manifestation of such exposure cannot be definitively established in the present study. To explore this, we assessed T-cell responses to 15 predicted SARS-CoV-2 N 15mer peptides against PBMCs from 16 study participants, to confirm their epitope status. At baseline, 57 N peptide responses met the predefined positivity threshold, originating from 11 of the 16 participants (68.8%) whose PBMCs were stimulated with the predicted N peptides (**Table 4**; **Supplementary Table 3**). Participant J028 responded to all 15 peptides, P084 responded to 12 peptides, P099 responded to 7 peptides and J189 to 6 peptides (**Table 4**).

**Table 4:** Vaccination strategy and number of positive N peptides per participant.

Participant ID	N peptide positivity per participant at:			
	Month 0	Month 3	Month 6	Month 9
Ad26.COV2.S-Boosted Participants				
J006	1	0	N/A	N/A
J023	5	4	0	0
J028	15	7	1	1
J040	0	0	N/A	N/A
J060	1	N/A	N/A	N/A
J076	1	0	N/A	N/A
J172	1	0	N/A	N/A
J189	6	4	0	1
BNT162b2-Boosted Participants				
P011	5	1	1	0
P060	0	4	1	0
P064	0	3	2	0
P079	0	1	0	0
P084	12	4	4	1
P085	0	6	0	0
P089	3	0	N/A	N/A
P099	7	0	N/A	N/A
Total positive peptides at each time point	57	34	9	3

Among all tested participants, N-specific IFN-γ responses demonstrated an overall decline from Month 0 through Month 6, with modest stabilization by Month 9 (**Figure 6**; **Supplementary Excel File 4**). Pairwise contrasts confirmed a non-significant reduction between Month 0 and Month 3, followed by significant decreases between Month 0 and Months 6 and 9, with large standardized effect sizes. No significant differences were detected between Months 3 and 9 or between Months 6 and 9, indicating relative stabilization of N-specific responses beyond six months. Sensitivity analyses showed a significant overall effect of time on total IFN-γ output but not on median per-peptide responses, suggesting that longitudinal changes reflect contraction of epitope breadth rather than attenuation of per-peptide response strength.

**Figure 6:**
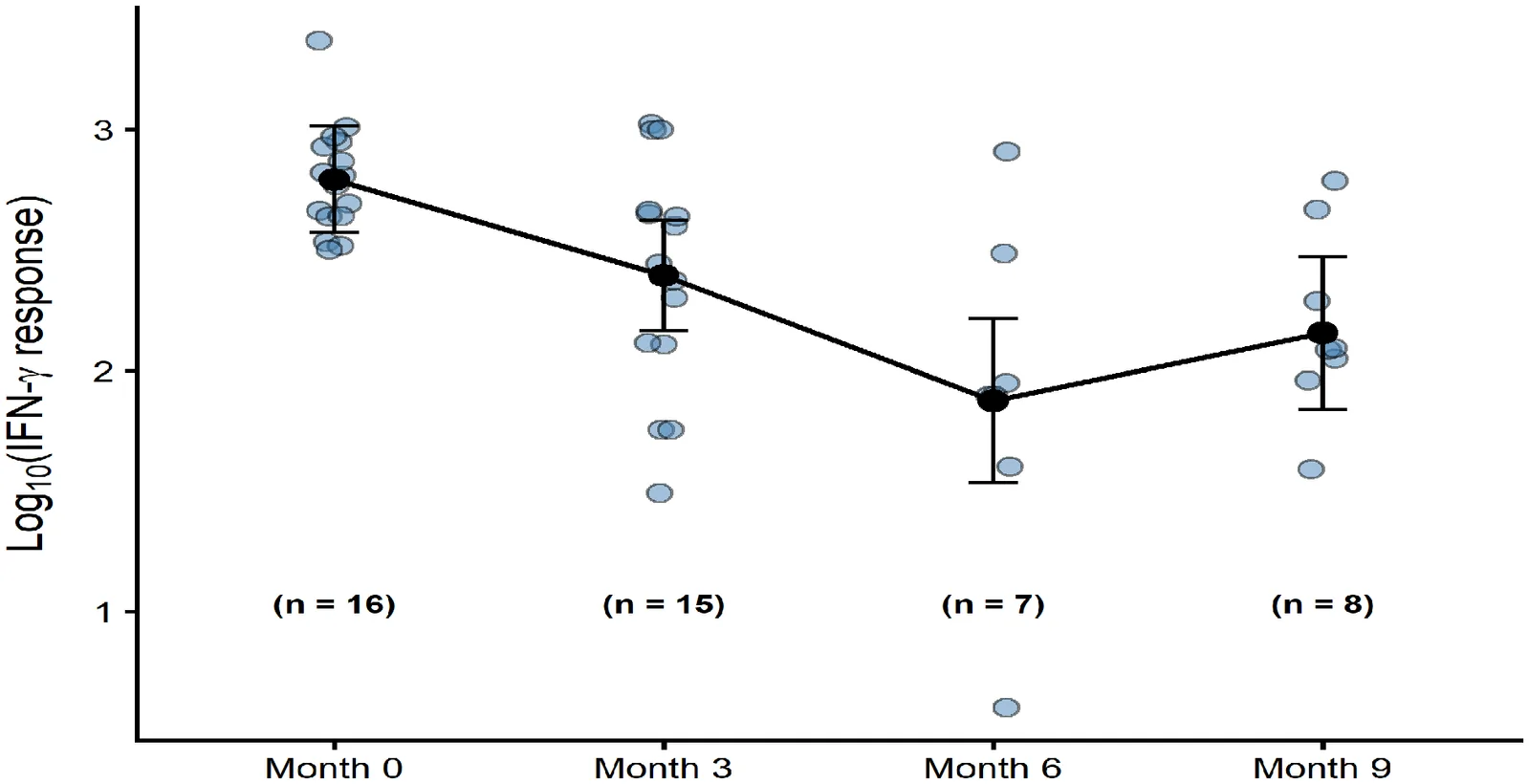
Temporal trend of N-specific T cell IFN-γ responses across the four sampling time points. Participant-level IFN-γ responses (log_10_-transformed) are shown at Months 0 (pre-booster), 3, 6, and 9 post-booster. The N-specific IFN-γ response at each time point represents the sum of the IFN-γ responses to all previously assessed N-peptide-specific reactions. Light blue dots represent individual participants with detectable responses at each time point. Black dots indicate marginal group means estimated from a linear mixed-effects model with participant as a random intercept. Error bars reflect 95% confidence intervals, and black lines connect group means to visualize temporal trends. Numbers below each time point indicate the number of participants contributing data (n). Analyses are restricted to persisting responses (total IFN-γ > 0), such that the absence of a response is not treated as zero. Estimated mean values at each time point are listed in the **Supplementary Excel File**.

Across all sampling points, two participants (J028 and P084) exhibited sustained N peptide reactivity, with P084 uniquely recognizing the same set of peptides at both Months 3 and 6. Three additional participants (P011, P060, and P064) maintained responses up to Month 6 (**Table 4**).

N-specific peptide recognition patterns also differed between the booster groups over time. The Ad26.COV2.S booster group demonstrated a higher frequency of positive peptide-specific responses at baseline and Month 9, whereas the BNT162b2 group showed greater recognition at Months 3 and 6. Despite these temporal differences, two N peptide regions—YGANKDGIIW (aa 121–135) and VTPSGTWLTY (aa 321–335)—were consistently recognized across all time points. Four additional peptides were recurrently recognized up to Month 6 (**Supplementary Table 3**). Again, there was significant heterogeneity in the magnitude of IFN-γ responses for the nine individuals who responded to the same N peptides across the four time points (**Supplementary Figure 4**).

## Discussion

This longitudinal study evaluated the durability and antigen specificity of SARS-CoV-2 S- and N-specific T-cell responses in a Ghanaian adult cohort following homologous and heterologous booster vaccination with BNT162b2 or Ad26.COV2.S vaccines. A defining feature of this cohort is that all participants were nucleocapsid-seropositive before boosting (Partey et al., 2026). The responses reported here therefore describe booster vaccination on a background of hybrid immunity rather than vaccine-induced immunity alone. Responses were measured by IFN-γ ELISpot against peptides selected via MHC class I restriction prediction to enrich for putative CD8^+^ epitopes as a functional readout of cellular immunity. Across the 9-month follow-up, both S- and N-specific T-cell responses declined primarily through contraction in the breadth of recognized epitopes rather than loss of per-epitope functional strength, with the residual response stabilizing beyond six months post-boost. Booster platform and vaccination strategy shaped this trajectory: BNT162b2 boosting produced higher early response magnitude, whereas homologous Ad26.COV2.S boosting sustained the broadest and most durable epitope recognition through Month 9. Focusing on the S-specific response, cumulative IFN-γ levels were higher among BNT162b2-boosted participants from baseline through Month 6, whereas Ad26.COV2.S recipients showed comparatively higher levels by Month 9 (**Figure 3**). Longitudinal modelling indicated that BNT162b2-induced responses underwent marked contraction of epitope breadth by six months, with per-epitope IFN-γ production showing relative stabilization thereafter, while Ad26.COV2.S-induced responses showed early stability followed by a similar pattern of breadth contraction and later stabilization. Overall, Ad26.COV2.S-boosted participants exhibited significantly broader S-specific T-cell responses than BNT162b2 recipients (**Table 3**; **Supplementary Table 2**) an advantage concentrated in the homologous Ad26→Ad26 subgroup, which contributed the highest proportion of persistent responses at Months 6 and 9.

These findings align with reports that mRNA platforms induce rapid antigen expression and robust early T-cell activation through efficient cytoplasmic translation and endogenous MHC I/II presentation (Pardi et al., 2018; Goel et al., 2021; Litvinova et al., 2023). However, the inherently transient nature of mRNA, coupled with interferon-induced RNA degradation, limits the duration of antigen availability, which may constrain prolonged restimulation of low-frequency or subdominant T-cell clones over time (Pardi et al., 2018; Litvinova et al., 2023). Although not directly tested in the present study, previous studies (Marchi et al., 2019; Provine and Klenerman, 2023) have demonstrated adenoviral vector platforms such as Ad26 is associated with more prolonged antigen expression and sustained innate and adaptive immune activation in some settings, potentially supporting extended maintenance of antigen-specific T-cell repertoires (Marchi et al., 2019; Provine and Klenerman, 2023).While not implying ongoing antigen priming at later time points, such prolonged antigen exposure early after boosting may favor broader initial recruitment and enhanced persistence of epitope-specific memory T cells. This framework provides a plausible biological explanation for the broader and more durable S-specific T-cell breadth observed in Ad26.COV2.S–boosted participants in this study.

Our results demonstrate a distinct immunological advantage following homologous Ad26.COV2.S boosting, with consistently broader recognition of S-specific epitopes and detectable responses persisting up to Month 9 in a subset of participants. This group also contributed the highest proportion of positive T-cell responses at Months 6 and 9 (**Supplementary Table 2**), suggesting enhanced durability of cellular immunity. The observed breadth may reflect prolonged *in vivo* antigen expression associated with adenoviral vector platforms, which can sustain antigen availability during early post-boost periods and enhance the likelihood of diverse antigen-presenting cell engagement. This extended presentation may promote recruitment of subdominant epitopes and contribute to the reshaping of immunodominance hierarchies, consistent with inflationary-like memory dynamics described for persistent antigen exposure (Lee et al., 2017; Hyun et al., 2023).

Importantly, our findings indicate a decoupling between response magnitude and epitope breadth. Although heterologous mRNA→Ad26 recipients exhibited higher IFN-γ levels at Month 3 compared to Ad26→Ad26 recipients (p = 0.19, **Supplementary Figure 2**), homologous Ad26→Ad26 vaccination was associated with significantly broader epitope recognition (p = 0.0001, **Supplementary Table 2**). This suggests that the quality of antigen presentation and T-cell priming may be more critical determinants of epitope diversity than peak effector magnitude alone. While anti-vector immunity is often proposed as a limitation of homologous adenoviral boosting, its impact appears context-dependent and may be mitigated in heterologous prime–boost regimens or incompletely inhibitory even in homologous Ad26 strategies. Notably, mRNA-based regimens showed higher early response rates at Month 3 (mRNA→Ad26: 71.4%, mRNA→mRNA: 70%, Ad26→mRNA: 53.9%) (**Supplementary Table 2**), yet this did not translate into sustained epitope breadth over time. Collectively, these findings suggest that Ad26.COV2.S boosting preferentially supports durable epitope diversity, which may enhance the likelihood of cross-variant T-cell recognition. However, it does not necessarily translate into improved protection on its own. Further studies integrating functional T-cell profiling and clinical outcomes are therefore required to define the protective relevance of these qualitative differences.

Additionally, no significant differences in IFN-γ activity or epitope recognition frequency were observed between homologous and heterologous subgroups in the BNT162b2 cohort. These findings may reflect limited statistical power rather than true immunological equivalence. More broadly, subgroup comparisons stratified by booster type and vaccination strategy are modestly powered, and any observed significance should be interpreted with caution. Validation in larger, well-stratified cohorts will be essential to confirm or refute these patterns.

Peptide mapping also revealed dynamic shifts in the immunodominance hierarchy over time. At baseline, the responses clustered around the NTD and fusion peptide regions (**Supplementary Table 1**). These structurally exposed domains have previously been shown to harbor conserved T cell epitopes across SARS-CoV-2 variants (Grifoni et al., 2021; Tarke et al., 2022). Following booster vaccination, recognition shifted toward the RBD and subdomain 1/2, with increased targeting of peptides such as YNYKLPDDFTGCVIA (aa 421–435) and PVAIHADQLTPTWRV (aa 621–635). This transition may reflect the recall of subdominant memory T cell clones not strongly activated during the primary response, or the neo-priming of naïve T cells driven by altered antigen routing and processing post-boost (Pennock et al., 2013; Sette et al., 2023). By Month 9, only nine peptides remained immunoreactive—three each from the NTD and fusion peptide regions, and one each from the RBD, SD1/2, and heptad repeat 2 (HR2). The persistence of NTD and fusion-derived epitopes underscores their structural stability and immunological resilience, supporting their inclusion in multi-epitope T cell–based vaccine designs (Tang et al., 2025). The rare persistence of a peptide from the HR2 domain is notable, as this region is largely buried in the prefusion S conformation, potentially limiting its accessibility for MHC presentation. Its immunogenicity in select individuals may reflect unique HLA binding affinities or exposure to post-fusion S conformations (Maltseva et al., 2024), warranting further investigation. Together, these findings suggest that next-generation vaccines aiming to elicit durable T-cell immunity should prioritize structurally conserved regions such as the NTD and fusion domains that resist epitope decay over time. At the same time, the shifting immunodominance observed post-booster highlights the potential for selective pressure on newly targeted regions such as the RBD, with implications for T cell immune escape in emerging variants. Including a broader range of conserved epitopes in vaccine constructs may help mitigate this risk by distributing immune pressure across multiple domains.

Furthermore, individual-level patterns such as those observed in participants J028 and J171 illustrate the potential for long-lived, cross-temporal T cell responses in certain vaccine recipients. This variability may reflect underlying differences in HLA haplotypes, prior antigen exposure, or intrinsic T cell repertoire diversity, factors known to influence T cell longevity and epitope specificity (Copley et al., 2021; Sette and Crotty, 2021; Braun et al., 2024).

Across the combined BNT162b2 and Ad26.COV2.S cohort, N-specific IFN-γ responses demonstrated an overall decline from Month 0 through Month 6, with modest stabilization by Month 9. Because all participants were nucleocapsid-seropositive at baseline and the administered vaccines encode the S protein alone, these responses represent infection-acquired cellular memory (Partey et al., 2026). Anti-nucleocapsid antibody levels remained stable across all four sampling timepoints, and no incident infection was detected, establishing that the contraction of N-specific T-cell responses occurred without renewed exposure to N antigen. The S- and N-specific compartments diverged over the first three months after boosting. S-specific responses were maintained across this interval (p = 0.976) and rose numerically in the Ad26.COV2.S group, whereas N-specific responses had already begun to decline. Because the booster delivered S antigen and no N antigen, this early divergence implies that spike-based vaccination does not sustain non-spike responses, consistent with observations in previous studies (Ahmed et al., 2022; Cai et al., 2023).

Although a modest increase in N-specific IFN-γ responses was observed in the Ad26.COV2.S booster group between Months 6 and 9, this trend did not reach statistical significance after adjustment, suggesting a stabilization rather than true recovery of N-specific cellular immunity at later time points. Because anti-nucleocapsid antibody levels remained stable throughout follow-up, intermittent subclinical re-exposure is unlikely to account for the minor longitudinal fluctuations observed in median per-peptide responses. These more probably reflect homeostatic proliferation of memory T cells, particularly effector memory subsets (Kato et al., 2018) or intrinsic assay variability (Allan-Blitz et al., 2021). Alternatively, the homeostatic proliferation of memory T cells, particularly effector memory subsets, may restore IFN-γ capacity following the resolution of inflammation in lymphoid tissues. Two regions in the N protein, YGANKDGIIW (aa 121–135) and VTPSGTWLTY (aa 321–335) were recognized at all four timepoints, by 5/16 and 4/16 participants at Month 0 and by a single participant at Month 9 respectively. The early and repeated recognition of the peptides, coupled with strong sequence conservation, makes them worth further evaluation in larger cohorts as candidate T-cell epitopes for multi-antigen vaccine design (Kumar et al., 2020; Tang et al., 2025).

This study has several limitations. First, although SARS-CoV-2 exposure status was characterized serologically at every sampling timepoint, the timing and clinical severity of the infections preceding enrolment could not be established. Pre-enrolment exposure history could therefore not be related to the magnitude or breadth of the responses observed. Second, although all participants received a booster at least six months after primary vaccination, the interval since the last vaccine dose varied considerably (6–24 months), potentially influencing memory recall responses and contributing to differences between booster groups. Third, epitope specificity was inferred from predicted 10-mer peptides nested within experimentally tested 15-mer sequences, and the absence of HLA typing prevented direct assessment of genotype-specific responses. Nevertheless, peptide selection was guided by broad HLA class I supertype coverage, and the longitudinal within-participant design enabled evaluation of response dynamics independent of baseline HLA variation. Finally, T-cell responses at Months 6 and 9 were assessed only for peptides positive at Month 3. This approach allowed longitudinal tracking of previously detected responses and conserved sample usage but may have biased estimates of changes in epitope breadth. In particular, delayed or newly emerging responses to peptides that were negative at Month 3 would not have been captured. Therefore, the observed decline in breadth should be interpreted as a contraction among the Month-3-positive epitopes that were longitudinally tracked, rather than definitive evidence of a global loss of SARS-CoV-2-specific T-cell epitope breadth. Future studies assessing the full peptide panel at each longitudinal time point will be important to distinguish true loss of breadth from the emergence of new or delayed responses. Despite these limitations, the study provides valuable insights into the durability, specificity, and heterogeneity of booster-induced T-cell immunity and highlights vaccine platform and booster strategy as important determinants of long-term cellular immune responses.

In conclusion, booster vaccination elicited distinct patterns of SARS-CoV-2–specific T-cell immunity, with homologous Ad26.COV2.S booster generating the broadest and most durable S-specific responses, while mRNA-based regimens produced the highest peak responder frequencies. Two N-derived epitopes, YGANKDGIIW (aa 121–135) and VTPSGTWLTY (aa 321–335), emerged as consistently immunodominant across all four time points and, together with epitopes in the NTD and fusion peptide regions of S, correspond to sites previously reported as sequence-conserved across SARS-CoV-2 variants. The evolving pattern of S epitope recognition following boosting suggests dynamic immunodominance and potential immune-driven selection, yet the persistence of responses to conserved epitopes despite viral evolution underscores their resilience. Collectively, these findings support prioritizing such epitopes, alongside independent confirmation of their cross-variant conservation, in future SARS-CoV-2 vaccine designs aimed at enhancing the breadth, durability, and robustness of cellular immunity.

## Data Availability

The original contributions presented in the study are included in the article/**Supplementary Material**. Further inquiries can be directed to the corresponding author.

## Supplementary material

Supplementary Figure 1 Longitudinal IFN-γ response profiles following Ad26.COV2.S **(A)** and BNT162b2-BioNTech **(B)** booster vaccination. Participant profiles were evaluated across the four time points (Months 0, 3, 6, and 9) following booster administration. For the Ad26.COV2.S booster **(A)**, 12 participants made responses to 19 different peptides and for the BNT162b2 **(B)**, 12 participants made responses to 15 different peptides. The presented data were restricted to instances where at least one participant maintained a robust longitudinal response record, defined as having available data for a minimum of three out of the four sampling time points. IFN-γ response levels are expressed as log[(SFU/10^6 PBMCs) + 1] to normalize variance and accommodate baseline values, with individual participant IDs represented by unique shapes to facilitate longitudinal tracking of each peptide response.

Supplementary Figure 2 Longitudinal trend of S-specific IFN-γ responses among Ad26.COV2.S-boosted participants, stratified by booster strategy. Boxplots show log_10_-transformed IFN-γ spot counts in response to SARS-CoV-2 spike peptide stimulation at baseline (Month 0) and at 3-, 6-, and 9-months post-booster among Ad26.COV2.S-boosted participants. Responses are stratified by booster strategy: homologous (viral vector→viral vector; orange) and heterologous (mRNA→Ad26; green). The S-specific IFN-γ response at each time point represents the sum of the IFN-γ responses to all previously assessed S-peptide-specific reactions. Each dot represents summed responses across spike peptides per individual. Group medians ( $\hat{µ}$_median_) are marked by dark red dots and labelled at each time point. Boxes indicate the median and interquartile range (IQR); whiskers extend to 1.5× IQR. Sample sizes (n) are shown below each group. P values were computed using the Wilcoxon rank-sum test.

Supplementary Figure 3 Longitudinal trend of S-specific IFN-γ responses among BNT162b2-boosted participants, stratified by booster strategy. Boxplots show log_10_-transformed IFN-γ spot counts in response to SARS-CoV-2 spike peptide stimulation at baseline (Month 0) and at 3-, 6-, and 9-months post-booster among BNT162b2-boosted participants. Responses are stratified by booster strategy: homologous (mRNA→mRNA; orange) and heterologous (viral vector→mRNA; green). The S-specific IFN-γ response at each time point represents the sum of the IFN-γ responses to all previously assessed S-peptide-specific reactions. Each dot represents summed responses across spike peptides per individual. Group medians ( $\hat{µ}$_median_) are marked by dark red dots and labelled at each time point. Boxes indicate the median and interquartile range (IQR); whiskers extend to 1.5× IQR. Sample sizes (n) are shown below each group. P values were computed separately for the paired groups in each month using the Wilcoxon rank-sum test.

Supplementary Figure 4 Longitudinal IFNγ response profiles to N peptides across the four sampling time points. Panel **(A)** represents the responses of 3 Ad26.COV2.S-vaccinated participants to 13 N peptides and Panel **(B)** represents the responses of 6 BNT162b2-BioNTech-vaccinated participants to 11 N peptides. The presented data was restricted to instances where at least one participant maintained a robust longitudinal record, defined as having available data for a minimum of three out of the four sampled time points. Response levels are expressed as log[(SFU/10^6 PBMCs) + 1] to normalize variance and accommodate baseline values. Individual participant IDs are represented by unique shapes to facilitate longitudinal tracking across all peptide facets.
