# Supplementary material for: T-cell recognition of SARS-CoV-2 antigen peptides following booster vaccination with mRNA and viral-vectored COVID-19 vaccines
Source: Front Cell Infect Microbiol. 2026 Oct 19;16:1947651. doi: 10.3389/fcimb.2026.1947651 (PMC13724500; doi:10.3389/fcimb.2026.1947651)
Supplement: Supplementary file 1 [file Table1.docx]

**Supplementary Table 1. Assay positivity of T-cell predicted Spike peptides over the four sampling timepoints**

| **Peptide Characteristics** | | | **Number of positive individuals** | | | |
| --- | --- | --- | --- | --- | --- | --- |
| **Predicted S Peptide** | **HLA haplotype** | **15mer Sequence ID** | **month 0** | **Month 3** | **Month 6** | **Month 9** |
| VYSSANNTF | HLA A24:02 | SGP030 (aa 136-160) | **4** | **6** | **2** | **1** |
| SSANNCTFEY | HLA A01:01 | SGP032 (aa 156-170) | **6** | **7** | **1** | **1** |
| YLQPRTFLLK | HLA A03:01 | SGP054 (aa 266-280) | **3** | **6** | **1** | **1** |
| ATRFASVYAW | HLA B58:01 | SGP069 (aa 341-355) | **4** | **6** | **1** | 0 |
| KLNDLCFTNV | HLA-A02:01 | SGP077 (aa 381-395) | **4** | **7** | **1** | 0 |
| RQIAPGQTGK | HLA A03:01 | SGP082 (aa 406-420) | **2** | **6** | **1** | **1** |
| KLPDDFTGCV | HLA-A02:01 | SGP085 (aa 421-435) | **4** | **8** | **1** | 0 |
| NLDSKVGGNY | HLA A01:01 | SGP088 (aa 436-450) | **3** | **2** | 0 | 0 |
| NTSNQVAVLY | HLA A01:01 | SGP121 (aa 601-615) | **4** | **4** | **1** | 0 |
| IHADQLTPTW | HLA B58:01 | SGP125 (aa 621-635) | **4** | **8** | **1** | **2** |
| TEILPVSMTK | HLA A03:01 | SGP145 (aa 721-735) | **3** | **2** | 0 | 0 |
| KQIYKTPPIK | HLA A03:01 | SGP157 (aa 781-795) | **2** | **3** | **1** | **1** |
| ILPDPSKPSK | HLA A03:01 | SGP161 (aa 801-815) | **4** | **4** | **1** | 0 |
| KRSFIEDLLF | HLA B58:01 | SGP163 (aa 811-825) | **6** | **5** | **1** | 0 |
| **LADAGFIKQY** | HLA A01:01 | SGP166 (aa 826-840) | **8** | **5** | **2** | **1** |
| LLTDEMIAQY | HLA A01:01 | SGP173 (aa 861-875) | **3** | **5** | **2** | **1** |
| VEAEVQIDRL | HLA B40:01 | SGP198 (aa 986-1000) | **4** | **4** | **1** | 0 |
| HVTYVPAQEK | HLA A03:01 | SGP213 (aa 1061-1075) | **1** | **2** | **1** | 0 |
| SLIDLQELGK | HLA A03:01 | SGP239 (aa 1191-1205) | **3** | **4** | **1** | 0 |
| LIDLQELGKY | HLA A01:01 | SGP240 (aa 1196-1210) | **2** | **5** | **1** | **1** |
| FDEDDSEPVL | HLA B40:01 | SGP251 (aa 1251-1265) | **4** | **5** | **2** | **0** |
| SEPVLKGVKL | HLA B07:02 | SGP252 (aa 1256-1270) | **3** | 0 | N/A | N/A |
| **Total positive peptides at each time point** | | | **81** | **104** | **23** | **10** |
|  | | |  |  |  |  |

Participants' samples collected at baseline (Month 0) and Month 3 were stimulated with all predicted peptides, but only peptides that tested positive at Month 3 were re-assessed for responses at Months 6 and 9
