# Supplementary material for: T-cell recognition of SARS-CoV-2 antigen peptides following booster vaccination with mRNA and viral-vectored COVID-19 vaccines
Source: Front Cell Infect Microbiol. 2026 Oct 19;16:1947651. doi: 10.3389/fcimb.2026.1947651 (PMC13724500; doi:10.3389/fcimb.2026.1947651)
Supplement: Supplementary file 2 [file Table2.docx]

**Supplementary Table 2**

| ***Number of Participants Showing Spike-Specific T Cell Positivity Over Time*** | | | | | | |
| --- | --- | --- | --- | --- | --- | --- |
| **Booster Group** | **Subgroup** | **n** | **Month 0** | **Month 3** | **Month 6** | **Month 9** |
| Ad26.COV2.S | Homologous | 16 | 9 (56.3%) | 8 (50%) | 5 (31.3%) | 2 (12.5%) |
|  | Heterologous | 7 | 1 (14.2%) | 5 (71.4%) | 2 (28.6%) | 0 (0%) |
|  | | | | | | |
| BNT162b2 | Homologous | 10 | 5 (50%) | 7 (70%) | 1 (10%) | 0 (0%) |
|  | Heterologous | 13 | 7 (53.9%) | 7 (53.9%) | 1 (7.7%) | 1 (7.7%) |

*Peptide responses are presented as counts and percentages [n (%)].*
