# Supplementary material for: T-cell recognition of SARS-CoV-2 antigen peptides following booster vaccination with mRNA and viral-vectored COVID-19 vaccines
Source: Front Cell Infect Microbiol. 2026 Oct 19;16:1947651. doi: 10.3389/fcimb.2026.1947651 (PMC13724500; doi:10.3389/fcimb.2026.1947651)
Supplement: Supplementary file 3 [file Table3.docx]

**Supplementary Table 3**

| ***Comparison of Spike Epitope Recognition Within Booster Groups*** | | | | |
| --- | --- | --- | --- | --- |
| **Ad26.COV2.S Booster Group** | | | | |
| Timepoint | Total (n=127) | Homologous (n=109) | Heterologous (n=18) | p-value |
| Month 0 | 35 (27.6%) | 33 (30.3%) | 2 (11.1%) | 0.007 |
| Month 3 | 70 (55.1%) | 57 (52.3%) | 13 (72.2%) | 0.0001 |
| Month 6 | 13 (10.2%) | 10 (9.2%) | 3 (16.7%) | 1.000 |
| Month 9 | 9 (7.1%) | 9 (8.2%) | 0 | 0.035 |
| **BNT162b2 Booster Group** | | | | |
| Timepoint | Total (n=91) | Homologous (n=34) | Heterologous (n=57) | p-value |
| Month 0 | 46 (50.5%) | 16 (47.0%) | 30 (52.6%) | 0.30 |
| Month 3 | 34 (37.4%) | 14 (41.2%) | 20 (35.1%) | 0.43 |
| Month 6 | 10 (11%) | 4 (11.8%) | 6 (10.5%) | 1.000 |
| Month 9 | 1 (1.1%) | 0 | 1 (1.8%) | 1.000 |

*Peptide responses are presented as counts and percentages [n (%)]. P-values were calculated using the Chi-square test of independence with Yates’ continuity correction. Fisher’s Exact Test was used when expected cell counts were below 5.*
