# Supplementary material for: T-cell recognition of SARS-CoV-2 antigen peptides following booster vaccination with mRNA and viral-vectored COVID-19 vaccines
Source: Front Cell Infect Microbiol. 2026 Oct 19;16:1947651. doi: 10.3389/fcimb.2026.1947651 (PMC13724500; doi:10.3389/fcimb.2026.1947651)
Supplement: Supplementary file 4 [file Table4.docx]

**Supplementary Table 4**

| A. ***Comparison of Positive Spike Peptide Recognition Between Booster Groups*** | | | | |
| --- | --- | --- | --- | --- |
| **Booster Group** | | | | |
| Timepoint | Total (n=218) | Ad26.COV2.S (n=127) | BNT162b2(n=91) | p-value |
| Month 0 | 81 (37.2%) | 35 (27.6%) | 46 (50.5%) | 0.75 |
| Month 3 | 104 (47.7%) | 70 (55.1%) | 34 (37.4%) | < 0.0001 |
| Month 6 | 23 (10.5%) | 13 (10.2%) | 10 (11%) | 0.16 |
| Month 9 | 10 (4.6%) | 9 (7.1%) | 1 (1.1%) | 0.017 |

*Peptide responses are presented as counts and percentages [n (%)]. P-values were calculated using the Chi-square test of independence with Yates’ continuity correction. Fisher’s Exact Test was used when expected cell counts were below 5.*
