# Supplementary material for: T-cell recognition of SARS-CoV-2 antigen peptides following booster vaccination with mRNA and viral-vectored COVID-19 vaccines
Source: Front Cell Infect Microbiol. 2026 Oct 19;16:1947651. doi: 10.3389/fcimb.2026.1947651 (PMC13724500; doi:10.3389/fcimb.2026.1947651)
Supplement: Supplementary file 5 [file Table5.docx]

**Supplementary Table 5**

| A. ***Comparison of Spike Epitope Recognition Based on Vaccination Strategy*** | | | | | | | |
| --- | --- | --- | --- | --- | --- | --- | --- |
| **Homologous-Boosted Participants** | | | | | | | |
| Timepoint | Total (n=143) | | Ad26.COV2.S (n=109) | | BNT162b2 (n=34) | p-value | |
| Month 0 | 49 (34.3%) | | 33 (30.3%) | | 16 (47.0%) | 0.26 | |
| Month 3 | 71 (49.6%) | | 57 (52.3%) | | 14 (41.2%) | 1.11 × 10⁻⁶ | |
| Month 6 | 14 (9.8%) | | 10 (9.2%) | | 4 (11.8%) | 0.42 | |
| Month 9 | 9 (6.3%) | | 9 (8.2%) | | 0 | 0.017 | |
| **Heterologous-Boosted Participants** | | | | | | | |
| Timepoint | Total (n=75) | Ad26.COV2.S (n=18) | | BNT162b2(n=57) | | | p-value |
| Month 0 | 32 (42.7%) | 2 (11.1%) | | 30 (52.6%) | | | 0.003 |
| Month 3 | 33 (44%) | 13 (72.2%) | | 20 (35.1%) | | | 0.89 |
| Month 6 | 9 (12%) | 3 (16.7%) | | 6 (10.5%) | | | 0.43 |
| Month 9 | 1 (1.3%) | 0 | | 1 (1.8%) | | | 1.00 |

*Peptide responses are presented as counts and percentages [n (%)]. P-values were calculated using the Chi-square test of independence with Yates’ continuity correction. Fisher’s Exact Test was used when expected cell counts were below 5.*
