# Supplementary material for: T-cell recognition of SARS-CoV-2 antigen peptides following booster vaccination with mRNA and viral-vectored COVID-19 vaccines
Source: Front Cell Infect Microbiol. 2026 Oct 19;16:1947651. doi: 10.3389/fcimb.2026.1947651 (PMC13724500; doi:10.3389/fcimb.2026.1947651)
Supplement: Supplementary file 6 [file Table6.docx]

**Supplementary Table 6. Assay positivity of T-cell Predicted Nucleocapsid peptides across the longitudinal timepoints**

| **Peptide Characteristics** | | | **Positivity at each time point** | | | |
| --- | --- | --- | --- | --- | --- | --- |
| **Predicted N sequence** | **HLA haplotype** | **Ref. Sequence ID** | **Month 0** | **Month 3** | **Month 6** | **Month 9** |
| RPQGLPNNTA | HLA B07:02 | np08 (aa 36-50) | **2** | **4** | 0 | 0 |
| KFPRGQGVPI | HLA B07:02 | np13 (aa 61-75) | **2** | **3** | **1** | 0 |
| FPRGQGVPIN | HLA B07:02 | np14 (aa 66-80) | **2** | **2** | **1** | 0 |
| NSSPDDQIGY | HLA A01:01 | np16 (aa 76-90) | **5** | **1** | 0 | 0 |
| LSPRWYFYYL | HLA B07:02 | np21 (aa 101-115) | **5** | **2** | 0 | 0 |
| GTGPEAGLPY | HLA A01:01 | np23 (aa 111-125) | **5** | **1** | 0 | **1** |
| **YGANKDGIIW** | **HLA B58:01** | **np25** (aa 121-135) | **5** | **3** | **1** | **1** |
| RNPANNAAIV | HLA B07:02 | np30 (aa 146-160) | **4** | 0 | N/A | N/A |
| QLPQGTTLPK | HLA A03:01 | np32 (aa 156-170) | **3** | **3** | 1 | 0 |
| KKPRQKRTAT | HLA B07:02 | np51 (aa 251-265) | **4** | **2** | 0 | 0 |
| QFAPSASAFF | HLA A24:02 | np62 (aa 306-320) | **4** | **3** | 0 | 0 |
| FGMSRIGMEV | HLA A02:01 | np63 (aa 311-325) | **5** | **1** | **1** | 0 |
| **VTPSGTWLTY** | **HLA A01:01** | **np65** (aa 321-335) | **4** | **5** | **3** | **1** |
| LLNKHIDAYK | HLA A03:01 | np71 (aa 351-365) | **3** | **2** | 0 | 0 |
| KTFPPTEPKK | HLA A03:01 | np72 (aa 356-370) | **4** | **2** | **1** | 0 |
| **Total positive peptides at each time point** | | | **57** | **34** | **9** | **3** |

*Participants' samples collected at baseline (Month 0) and Month 3 were stimulated with all predicted peptides, but only peptides that tested positive at Month 3 were re-assessed for responses at Months 6 and 9.*
